# The Jacksonian Prize

**Published:** 1836-07

**Authors:** 


THE JACKSONIAN PRIZE.

The Jacksonian Prize for the year 1835, by the College of Surgeons, London, has been adjudged to *Frederick Ryland, Esq.* of Birmingham, for a disputation on "Injuries and Diseases of the Larynx and Trachea, and their Treatment."

